# Association of fine particulate matter exposure with acute noncardiovascular critical illnesses and in-hospital outcomes in patients receiving intensive cardiac care

**DOI:** 10.1186/s12889-020-08758-7

**Published:** 2020-05-01

**Authors:** Fei Chen, Qi Liu, Baotao Huang, Fangyang Huang, Yiming Li, Yong Peng, Mao Chen

**Affiliations:** 1 Department of Cardiology, West China Hospital, Sichuan University, No.37 Guoxue Alley, Chengdu, 610041 PR China; 2 West China School of Medicine, Sichuan University, Chengdu, China

**Keywords:** Fine particulate matter, Acute noncardiovascular critical illness, Acute cardiac care, Prognosis

## Abstract

**Background:**

The effect of short-term exposure to fine particulate matter (PM_2.5_) on the incidence of acute noncardiovascular critical illnesses (ANCIs) and clinical outcomes is unknown in patients with acute cardiovascular diseases.

**Methods:**

We conducted a retrospective study in 2337 admissions to an intensive cardiac care unit (ICCU) from June 2016 to May 2017. We used the 2-day average PM_2.5_ concentration before ICCU admission to estimate the individual exposure level, and patients were divided into 3 groups according to the concentration tertiles. Major ANCI was defined as the composite of acute respiratory failure, acute kidney injury, gastrointestinal hemorrhage, or sepsis. The primary endpoint was all-cause death or discharge against medical advice in extremely critical condition.

**Results:**

During the 12-month study period, the annual median concentration of PM_2.5_ in Chengdu, China was 48 μg/m^3^ (IQR, 33–77 μg/m^3^). More than 20% of admissions were complicated by major ANCI, and the primary endpoints occurred in 7.6% of patients during their hospitalization. The association of short-term PM_2.5_ exposure levels with the incidence of acute respiratory failure (adjusted *OR* [odds ratio] =1.31, 95% *CI* [confidence interval]1.12–1.54) and acute kidney injury (adjusted *OR* = 1.20, 95% *CI* 1.02–1.41) showed a significant trend. Additionally, there were numerically more cases of sepsis (adjusted *OR* = 1.21, 95% *CI* 0.92–1.60) and gastrointestinal hemorrhage (adjusted *OR* = 1.29, 95% *CI* 0.94–1.77) in patients with higher exposure levels. After further multivariable adjustment, short-term PM_2.5_ exposure levels were still significantly associated with incident major ANCI (adjusted *OR* = 1.32, 95% *CI* 1.12–1.56), as well as a higher incidence of the primary endpoint (adjusted *OR* = 1.52, 95% *CI* 1.09–2.12).

**Conclusion:**

Short-term PM_2.5_ exposure before ICCU admission was associated with an increased risk of incident major ANCI and worse in-hospital outcomes in patients receiving intensive cardiac care.

## Background

During the past two decades, the body of evidence regarding the hazardous effect of ambient air pollution on public health has grown substantially [1, 2]. The Global Burden of Disease (GBD) Study 2016 showed that ambient particulate matter had become the sixth-leading risk factor for disability-adjusted life-years globally, and 11.1% of all death could be attributed to it in China [3]. Among various particulate matters, fine particulate matter, of which the aerodynamic diameter is less than 2.5 μm (PM_2.5_), is widely considered as the predominant pollutant [3–5]. Moreover, many observational studies have found that the risk of acute cardiovascular events and mortality was significantly associated with short-term PM_2.5_ exposure in the general population [6–8].

In recent years, researchers also noted that acute noncardiovascular critical illnesses (ANCI) were prevalent and correlated with increased mortality in patients with acute cardiovascular diseases admitted to intensive cardiac care units (ICCUs), who tended to be elderly and complicated by numerous chronic comorbidities [9, 10]. Theoretically, the effect of short-term PM_2.5_ exposure on these susceptible patients who need intensive cardiac care is unlikely to be limited to the cardiovascular system [2], hence it is interesting whether short-term PM_2.5_ exposure is correlated with the risk of incident ANCI in these patients. However, no studies to date have been conducted to explore such a correlation.

We tested the hypothesis that short-term exposure to ambient fine particulate matter is associated with an increased risk of incident major ANCI and worse clinical outcomes in ICCU patients.

## Methods

### Study population

We studied consecutive admissions to the ICCU at West China Hospital affiliated to Sichuan University, an academic tertiary care center located in Chengdu, China, from June 2016 to May 2017. In our hospital, patients with cardiovascular diseases who require acute cardiac care but do not require unscheduled surgery or postsurgical management, are admitted to the ICCU. All the admissions to our ICCU during the study period were eligible for screen (*n* = 2873). We excluded admissions of patients without cardiovascular diseases (*n* = 6), patients with an ICCU stay lasting less than 4 h after scheduled interventional procedures (*n* = 150), and admissions of patients who were younger than 16 years old (*n* = 139). If a patient was ever admitted to the ICCU more than once during the same hospital stay, his/her admissions not meeting the exclusion criteria mentioned above were combined into one (296 into 143). Further, we removed the non-first admissions for patients with multiple ICCU admissions during the study period (*n* = 88).

We systematically queried the electronic medical records and carefully reviewed all available information to obtain demographics, vital signs, laboratory data, cardiovascular and noncardiovascular diagnoses, therapeutic interventions, length of stay (LOS), and clinical outcomes. The type of admission was categorized as medical if no relevant interventional procedure was performed in the 7 days before or after ICCU admission. The local institutional review board approved this study, with a waiver of informed consent.

### Environmental data

We obtained daily PM_2.5_ data from the China National Environmental Monitoring Centre (http://www.cnemc.cn/) and China Air Quality Online Monitoring and Analysis Platform (https://www.aqistudy.cn). PM_2.5_ concentrations were measured using methods based on technical specifications issued by the Ministry of Environmental Protection in state-controlled monitoring sites. The 24-h mean PM_2.5_ concentration was simply averaged from all valid sites in this city. We used the average of 24-h mean PM_2.5_ concentration of the preceding day and the current day of ICCU admission to estimate the individual short-term exposure level. To adjust the impact of weather conditions, we obtained daily mean temperature and relative humidity from the National Meteorological Information Center (http://data.cma.cn/).

### Complexity and severity of illnesses

To assess the complexity of chronic illnesses of each ICCU admission, we calculated the Charlson Comorbidity Index (CCI), which is a weighted index that takes into account the number and the seriousness of comorbid disease [11]. Meanwhile, we also adopted the Oxford Acute Severity of Illness Score (OASIS) to evaluate acute severity of illnesses. OASIS includes 10 variables that can be easily captured, such as vital signs and mechanical ventilation status, and the final OASIS is the sum of the worst score of all the components across the first 24 h after ICCU admission [12].

### Key variable definitions

The composite of acute respiratory failure, acute kidney injury, gastrointestinal hemorrhage, or sepsis was defined as major ANCI, based on their prevalence and odds ratios for mortality reported by previous ICCU studies [9, 10]. The primary endpoint was the composite of all-cause death or discharge against medical advice (DAMA) in extremely critical condition, defined as the composite of circulatory shock, electrical instability requiring emergency medical interventions, or acute respiratory failure with the need for mechanical ventilation. The DAMA in extremely critical condition of critically ill patients is a relatively common phenomenon in China for a variety of reasons, including specific traditional prohibitions, financial concerns, and the lack of social support, etc., and those patients often suffer death shortly. Compared with all-cause death, the composite primary endpoint may reflect clinical outcomes of the study population more accurately.

### Statistical analysis

Based on the tertiles of the 2-day average concentration of PM_2.5_ before ICCU admission, patients were divided into low-exposure (≤36 μg/m^3^), medium-exposure (37-63 μg/m^3^), and high-exposure (≥64 μg/m^3^) groups. Baseline characteristics, major therapeutic interventions, and clinical outcomes were compared among patient groups. For continuous variables, we calculated means ± standard deviation or median (interquartile range, IQR), and the differences were tested using One-way Analysis of Variance or Kruskal–Wallis Tests, respectively. For categorical variables, we calculated counts and percentages, and Linear-by-Linear Association Chi-Square Tests were used to assess tendency changes across patient groups. To detect odds ratios for important characteristics according to short-term PM_2.5_ exposure levels in study population, we utilized Multivariate Binary Logistic Regression Models, controlling for mean temperature (per 5 °C increase) and relative humidity (per 10% increase). To explore the predictors of the incidence of the primary endpoint, we developed a Multivariate Binary Logistic Regression Model, adjusting for the 2-day average concentration of PM_2.5_ and weather conditions before ICCU admission, demographics, complexity and severity of illnesses, cardiogenic shock, ventricular arrhythmia, and major ANCI. A two-sided *P* value of less than 0.05 was considered as statistically significant. We performed all the statistical analyses in SPSS software (version 24.0).

## Results

A total of 2337 ICCU admissions were included in this study. The mean age of the patients was 65.6 ± 14.2 years, and male patients accounted for 68.0%. Most patients were admitted via emergency department (68.6%), and approximately 80% (77.8%) of patients underwent interventional procedures, in particular, nearly one-third (31.5%) of patients underwent unscheduled procedures.

During the 12-month study period, the annual median concentration of PM_2.5_ in Chengdu, China was 48 μg/m^3^ (IQR, 33–77 μg/m^3^), which was much higher than the theoretical minimum risk exposure level (2.4–5.9 μg/m^3^) defined by the GBD Study 2016 [3]. The concentration was also significantly higher than the levels (13.8–27.7 μg/m^3^) reported by epidemiological studies conducted in some developed countries [13, 14].

**Fig. 1:**
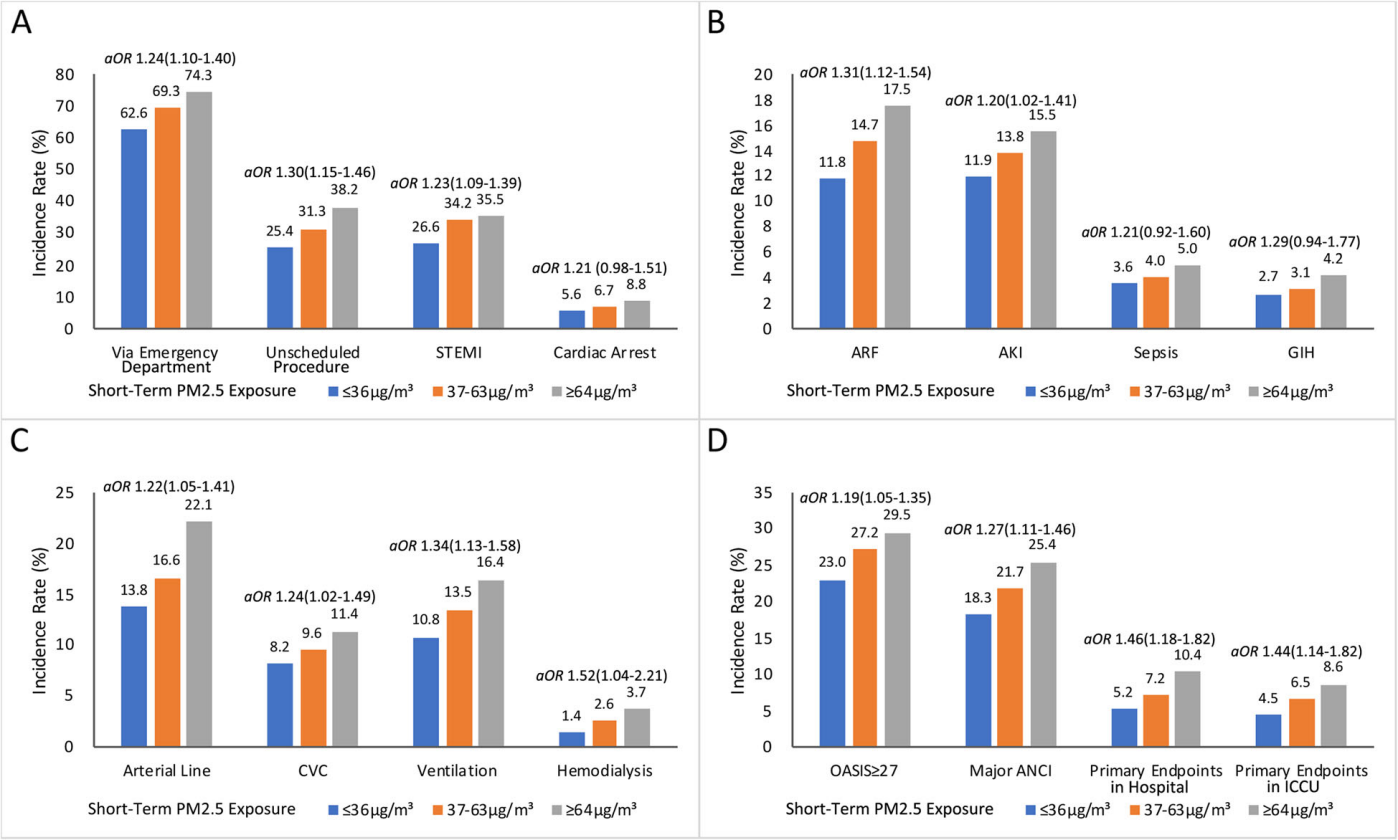
Adjusted odds ratios for important characteristics according to short-term PM_2.5_ exposure levels before ICCU admissions. Adjusted odds ratios (aORs) were estimated by logistic regression models, controlling for mean temperature (per 5 °C increase) and relative humidity (per 10% increase). Short-term PM_2.5_ exposure, the 2-day average PM_2.5_ concentration before ICCU admission. PM_2.5_, particulate matter with an aerodynamic diameter less than 2.5 μm; STEMI, ST-segment elevation myocardial infarction; ARF, acute respiratory failure; AKI, acute kidney injury; GIH, gastrointestinal hemorrhage; CVC, central venous catheter; OASIS, Oxford Acute Severity of Illness Score; ANCI, major acute noncardiovascular critical illness, defined as the composite of acute respiratory failure, acute kidney injury, sepsis, or gastrointestinal hemorrhage; Primary endpoint, the composite of all-cause death or discharge against medical advice in extremely critical condition

The baseline characteristics of the patients stratified by the 2-day average PM_2.5_ exposure levels before ICCU admission are presented in Table 1. There were differences in the source or type of admission, acute coronary syndrome (ACS), incident cardiac arrest, the rate of major ANCI, and acute severity of illnesses among patient groups. Even after controlling for weather conditions, the correlation of short-term PM_2.5_ exposure with admission via emergency department (*aOR* [adjusted odds ratio] = 1.24, 95%*CI* [confidence interval] 1.10–1.40), admission for ST-segment elevation myocardial infarction (STEMI) (*aOR* = 1.23, 95% *CI* 1.09–1.39), undergoing unscheduled procedures (*aOR* = 1.30, 95% *CI* 1.15–1.46), and the incidence of acute respiratory failure (*aOR* = 1.31, 95% *CI* 1.12–1.54) or acute kidney injury (*aOR* = 1.20, 95% *CI* 1.02–1.41), showed a significant trend with increasing exposure levels (Fig. 1, panel a and b). Additionally, there were numerically more cases of cardiac arrest (*aOR* = 1.21, 95% *CI* 0.98–1.51), sepsis (*aOR* = 1.21, 95%*CI* 0.92–1.60), and gastrointestinal hemorrhage (*aOR* = 1.29, 95% *CI* 0.94–1.77) in patients with higher PM_2.5_ exposure (Fig. 1, panel a and b). After further adjusting for age, CCI, cardiogenic shock, ventricular arrhythmia, and weather conditions, the incidence of major ANCI was still significantly correlated with short-term PM_2.5_ exposure levels (*aOR* = 1.32, 95% *CI* 1.12–1.56).

**Table 1:** Baseline characteristics of ICCU admission

	The 2-day average concentration of PM_**2.5**_ before ICCU admission			
Characteristic	≤36 μg/m^**3**^	37-63 μg/m^**3**^	≥64 μg/m^**3**^	***P*** value
	(*N* = 804)	(*N* = 773)	(*N* = 760)	
Age, yrs	65.9 ± 14.0	64.9 ± 14.6	65.8 ± 13.9	0.316
Age ≥ 65 yrs	473(58.8)	434(56.1)	446(58.7)	0.938
Male, n (%)	558(69.4)	531(68.7)	500(65.8)	0.128
Source of admission, n (%)				
Department of Emergency	503(62.6)	536(69.3)	565(74.3)	< 0.001
General Ward of Cardiology	254(31.6)	201(26.0)	165(21.7)	< 0.001
Other Departments	47(5.8)	36(4.7)	30(3.9)	0.080
Type of admission, n (%)				
Medical	176(21.9)	164(21.2)	178(23.4)	0.474
Scheduled procedure	424(52.7)	367(47.5)	292(38.4)	< 0.001
Unscheduled procedure	204(25.4)	242(31.3)	290(38.2)	< 0.001
Vital signs				
Temperature, °C	36.4 ± 0.8	36.4 ± 0.7	36.2 ± 2.7	0.208
Respiratory rate, per min	20.4 ± 3.5	20.3 ± 3.1	20.2 ± 4.5	0.745
Heart rates, beats/min	79.3 ± 21.5	79.8 ± 20.5	80.6 ± 22.4	0.467
Systolic blood pressure, mmHg	125.7 ± 45.0	124.0 ± 42.5	125.5 ± 45.8	0.717
Diastolic blood pressure, mmHg	72.6 ± 15.8	72.7 ± 15.8	72.6 ± 17.4	0.988
Charlson Comorbidity Index	2.3 ± 1.8	2.3 ± 1.9	2.4 ± 1.9	0.198
Oxford Acute Severity of Illness Score	21.5 ± 9.8	22.4 ± 10.7	23.7 ± 10.7	< 0.001
**Cardiovascular comorbidities, n (%)**				
Ischemic heart disease	578(71.9)	587(75.9)	548(72.1)	0.898
ACS	434(54.0)	468(60.5)	469(61.7)	0.002
STEMI	214(26.6)	264(34.2)	270(35.5)	< 0.001
NSTEMI	114(14.2)	123(15.9)	120(15.8)	0.371
UA	106(13.2)	81(10.5)	79(10.4)	0.080
SIHD	137(17.0)	112(14.5)	75(9.9)	< 0.001
MI mechanical complications	14(1.7)	10(1.3)	13(1.7)	0.951
Hypertension	432(53.7)	391(50.6)	388(51.1)	0.283
Myocardial disease	70(8.7)	65(8.4)	64(8.4)	0.838
Valvar heart disease	298(37.1)	285(36.9)	268(35.3)	0.463
Heart failure	472(58.7)	458(59.2)	460(60.5)	0.465
NYHA functional classification				
I	260(32.3)	250(32.3)	266(35.0)	0.268
II-III	291(36.2)	266(34.4)	264(34.7)	0.541
IV	167(20.8)	179(23.2)	180(23.7)	0.166
Cardiac shock	53(6.6)	64(8.3)	59(7.8)	0.372
Cardiac arrest	45(5.6)	52(6.7)	67(8.8)	0.013
Atrial arrhythmia	140(17.4)	130(16.8)	142(18.7)	0.517
Ventricular arrhythmia	63(7.8)	45(5.8)	51(6.7)	0.366
Bradycardia	92(11.4)	90(11.6)	68(8.9)	0.114
Congenital heart disease	13(1.6)	13(1.7)	14(1.8)	0.733
Pericardial disease	10(1.2)	9(1.2)	8(1.1)	0.724
Aortic disease	6(0.7)	13(1.7)	14(1.8)	0.065
Acute aortic syndrome	2(0.2)	3(0.4)	6(0.8)	0.120
Cerebrovascular disease	75(9.3)	55(7.1)	78(10.3)	0.539
Acute stroke/TIA	9(1.1)	5(0.6)	12(1.6)	0.400
Peripheral arterial disease	18(2.2)	14(1.8)	18(2.4)	0.869
Venous thromboembolism	11(1.4)	7(0.9)	11(1.4)	0.901
**Noncardiovascular comorbidities, n (%)**				
Tobacco abuse	279(34.7)	275(35.6)	245(32.2)	0.312
Alcohol abuse	56(7.0)	70(9.1)	57(7.5)	0.676
Diabetes mellitus	237(29.5)	210(27.2)	212(27.9)	0.479
Chronic kidney disease	155(19.3)	127(16.4)	153(20.1)	0.688
Hypercholesterolemia	118(14.7)	122(15.8)	139(18.3)	0.054
Chronic lung disease	120(14.9)	102(13.2)	101(13.3)	0.344
Malignancy	30(3.7)	25(3.2)	23(3.0)	0.436
Thyroid Disease	17(2.1)	8(1.0)	20(2.6)	0.479
Connective tissue disease	7(0.9)	9(1.2)	10(1.3)	0.400
Gout	23(2.9)	29(3.8)	19(2.5)	0.695
Moderate/severe anemia	76(9.5)	67(8.7)	75(10.0)	0.802
Hepatic dysfunction	56(7.0)	75(9.7)	70(9.2)	0.109
Dyskalemia	216(26.9)	232(30.0)	228(30.0)	0.168
Pneumonia/LRTI	214(26.6)	217(28.1)	231(30.4)	0.908
Major acute nonCV critical illnesses	147(18.3)	168(21.7)	193(25.4)	0.001
Acute respiratory failure	95(11.8)	114(14.7)	133(17.5)	0.001
Acute kidney injury	96(11.9)	107(13.8)	118(15.5)	0.039
Sepsis	29(3.6)	31(4.0)	38(5.0)	0.171
Gastrointestinal hemorrhage	22(2.7)	24(3.1)	32(4.2)	0.106

Data are expressed as means ± standard variation or counts and percentage, as appropriate

*Abbreviation*: *PM*_*2.5*_ particulate matter with an aerodynamic diameter less than 2.5 μm, *ACS* acute coronary syndrome, *STEMI* ST-segment elevation myocardial infarction, *NSTEMI* non-ST-segment elevation myocardial elevation, *UA* unstable angina, *SIHD* stable ischemic heart disease, *MI* myocardial infarction, *TIA* transient ischemic attack, *CV* cardiovascular, *LRTI* low respiratory tract infection

Major therapeutic characteristics of the patients are shown in Table 2. Consistent with the increasing trend of major ANCI, patients with higher PM_2.5_ exposure were more likely to receive invasive hemodynamic monitoring, such as arterial line (*aOR* = 1.22, 95% *CI* 1.05–1.41) and central venous catheter (*aOR* = 1.24, 95% *CI* 1.02–1.49), as well as mechanical ventilation (*aOR* = 1.34, 95% *CI* 1.13–1.58) and hemodialysis (*aOR* = 1.52, 95% *CI* 1.04–2.21) after considering weather conditions (Fig. 1, panel c).

**Table 2:** Major therapeutic characteristics of ICCU admission

	The 2-day average concentration of PM_**2.5**_ before ICCU admission			
Characteristic	≤36 μg/m^**3**^	37-63 μg/m^**3**^	≥64 μg/m^**3**^	***P*** value
	(*N* = 804)	(*N* = 773)	(*N* = 760)	
Percutaneous coronary intervention	446(55.5)	462(59.8)	433(57.0)	0.531
Temporary pacemaker	78(9.7)	72(9.3)	60(7.9)	0.214
Conventional pacemaker	46(5.7)	41(5.3)	24(3.2)	0.018
ICD	7(0.9)	4(0.5)	4(0.5)	0.389
CRT-P/D	3(0.4)	5(0.6)	3(0.4)	0.939
Ablation for tachycardia	43(5.3)	20(2.6)	25(3.3)	0.030
LAA closure	16(2.0)	2(0.3)	13(1.7)	0.593
TAVI or BAV	18(2.2)	10(1.3)	23(3.0)	0.302
Interventional therapy for CHD	6(0.7)	8(1.0)	5(0.7)	0.856
Intra-aortic balloon pump	28(3.5)	32(4.1)	27(3.6)	0.932
Inotropes or vasopressors	90(11.2)	115(14.9)	107(14.1)	0.089
Arterial line	111(13.8)	128(16.6)	168(22.1)	< 0.001
Central venous catheter	66(8.2)	74(9.6)	87(11.4)	0.031
Hemodialysis	11(1.4)	20(2.6)	28(3.7)	0.004
Mechanical ventilation	87(10.8)	104(13.5)	125(16.4)	0.001

Data are expressed as counts and percentage

*Abbreviation*: *PM*_*2.5*_ particulate matter with an aerodynamic diameter less than 2.5 μm, *ICD* implantable cardioverter defibrillator, *CRT-P/D* cardiac resynchronization therapy and pacemaker or defibrillator, *LAA closure* left atrial appendage closure, *TAVI* transcatheter aortic implantation, *BAV* balloon aortic valvuloplasty, *CHD* congenital heart disease

Median LOS in ICCU and hospital were 1.1 days (IQR, 0.8–2.6 days) and 6.3 days (IQR, 3.8–10.9 days), respectively. The overall rate of primary endpoint was 7.6% during hospitalization of study patients, with 6.5% occurring in ICCU. Clinical outcomes were considerably different among patient groups (Table 3). In accordance with the larger proportion of greater OASIS and more major ANCI in patients with higher PM_2.5_ exposure, primary endpoint (*aOR* = 1.46, 95%*CI* 1.18–1.82), as well as that occurring in ICCU (*aOR* = 1.44, 95% *CI* 1.14–1.82), were more frequent in these patients after controlling for weather conditions (Fig. 1, panel d).

**Table 3:** Clinical outcomes

	The 2-day average concentration of PM_**2.5**_ before ICCU admission			
Outcomes	≤36 μg/m^**3**^	37-63 μg/m^**3**^	≥64 μg/m^**3**^	***P*** value
	(*N* = 804)	(*N* = 773)	(*N* = 760)	
Primary endpoint	42(5.2)	56(7.2)	79(10.4)	< 0.001
DAMA from hospital in ECC	20(2.5)	24(3.1)	39(5.1)	0.005
DAMA from ICCU in ECC	15(1.9)	20(2.6)	32(4.2)	0.006
All-cause Death in hospital	22(2.7)	32(4.1)	40(5.3)	0.011
All-cause death in ICCU	21(2.6)	30(3.9)	33(4.3)	0.065

Data are expressed as median (interquartile) or counts (percentage), as appropriate

*Abbreviation*: *PM*_*2.5*_ particulate matter with an aerodynamic diameter less than 2.5 μm, *DAMA* discharge against medical advice, *ECC* extremely critical condition, define as the composite of circulatory shock, electrical instability requiring emergency interventions, or acute respiratory failure with the need for mechanical ventilation

**Fig. 2:**
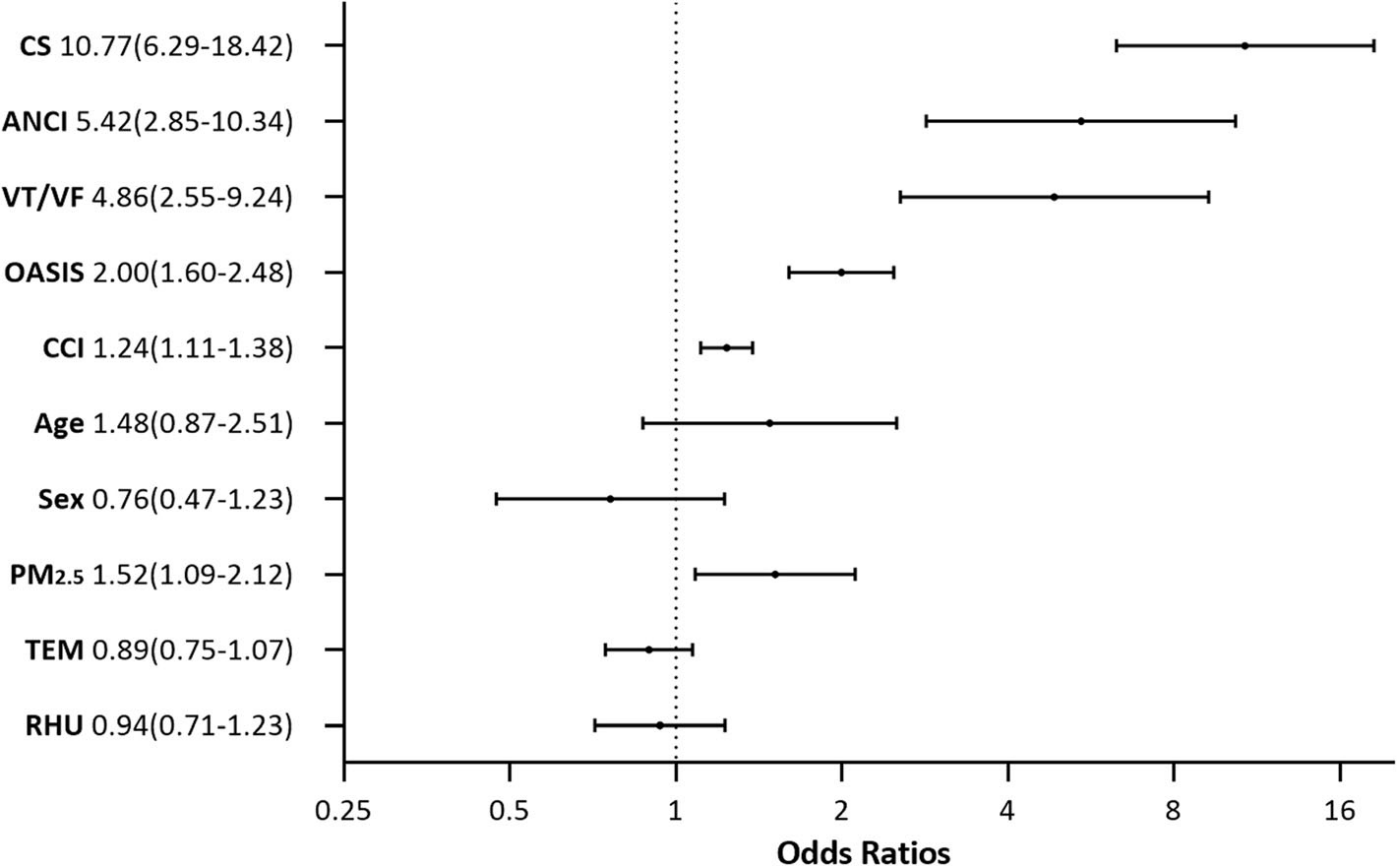
Predictor of in-hospital outcomes in ICCU patients. Odds ratios (ORs) for the primary endpoint were estimated by multivariate logistic regression model. CS, cardiogenic shock; ANCI, major acute noncardiovascular critical illness, defined as the composite of acute respiratory failure, acute kidney injury, sepsis, or gastrointestinal hemorrhage; VT/VF, ventricular tachycardia or fibrillation; OASIS, Oxford acute severity of illnesses Score, entering the model with per 10 scores increase; CCI, Charlson Comorbidity Index; Age, greater than or equal to 65 years; PM_2.5_, particulate matter with an aerodynamic diameter less than 2.5 μm, entering the model with per tertile increase of the 2-day average concentration before ICCU admission; TEM, mean temperature, entering the model with per 5 °C increase; RHU, relative humidity, entering the model with per 10% increase

The association between multiple predictors and in-hospital outcomes is displayed in Fig. 2. After further multivariable adjustment, short-term PM_2.5_ exposure levels before ICCU admission were still strongly associated with a higher incidence of the primary endpoint (*aOR* = 1.52, 95% *CI* 1.09–2.12, *P* = 0.015). However, the correlation between short-term PM_2.5_ exposure levels and primary endpoints occurring in ICCU was no longer statistically significant after the adjustment (*aOR* = 1.44, 95% *CI* 0.98–2.13, *P* = 0.067), possibly because of less statistical power due to fewer events occurring in ICCU.

## Discussion

In an academic tertiary care center, we investigated 2337 patients admitted to the ICCU over the course of one year, and found that short-term PM_2.5_ exposure before ICCU admission was associated with an increased risk of incident major ANCI (especially acute respiratory failure and acute kidney injury), greater acute severity of illnesses, increased need for advanced monitoring and therapeutic devices, as well as worse clinical outcomes. To our knowledge, this is the first study to report the short-term effect of ambient air pollution on patients who need acute cardiac care.

A large number of time-series studies have demonstrated that short-term PM_2.5_ exposure was associated with increased risk for near-term myocardial infarction, cardiac arrest, and mortality in the general population, although there was heterogeneity in the effect estimates in different studies [6–8]. Our data were mostly in agreement with previous studies. We also found that the increased risk of STEMI seemed to be more pronounced than that of other types of ACS, though admission bias could not be excluded as a contributing factor. Possible biological mechanisms contributing to the acute cardiovascular effect of PM_2.5_ exposure have been well described. Three broad intermediary pathways, including systemic oxidative stress and inflammation, autonomic imbalance favoring sympathetic activation, and potential direct actions of particulate matters reaching the systemic circulation, as well as the subsequent specific biological responses (e.g., endothelial dysfunction, vasoconstriction, plaque vulnerability, decreased heart rate variability, etc.), have been proposed to underlie cardiovascular events following short-term PM_2.5_ exposure [15, 16]. These underlying mechanisms may be primarily responsible for the difference in cardiovascular events in vulnerable ICCU patients exposed to variant PM_2.5_ levels.

We also found that the incidence of major ANCI was more prevalent in ICCU patients with higher short-term PM_2.5_ exposure. Thus far, studies about the health effect of ambient fine particulate matter have focused on the general population, while there have been no investigations of this effect in individuals requiring acute cardiac care. Due to the aging population and increasing chronic comorbidities, the vulnerability of these susceptible individuals tends to be more significant than that of the general population [17]. Consequently, these patients may suffer a larger clinically meaningful impact of short-term PM_2.5_ exposure that is not limited to the cardiovascular system. On the other hand, some studies have reported that ANCI is common and associates with increased mortality risk in contemporary ICCU settings [9, 10]. In consideration of a possible multisystemic consequence following short-term PM_2.5_ exposure and the meaningfulness of ANCI, it is crucial to explore whether short-term PM_2.5_ exposure is associated with the incidence of major ANCI. Our findings confirm the existence of this association.

Previous studies have linked short-term PM_2.5_ exposure to the increased risk of decreased lung function [18, 19] and acute respiratory failure [20, 21] in apparently healthy individuals. The mechanism suggested to explain this acute health effect is the acute airway response caused by activation of inflammatory pathways and small airway constriction owing to the chemical constituents of inhaled fine particulates [18, 21]. For vulnerable ICCU patients, the poorer physiological and pulmonary reserve could result in a more pronounced decrease in lung function following short-term PM_2.5_ exposure. This in turn may increase the risk of incident acute respiratory failure. Furthermore, some studies found that one-year PM_2.5_ exposure was associated with lower renal function in the general population [22, 23]. Although no human studies have ever investigated whether short-term PM2.5 exposure is linked to the incidence of acute kidney injury, some animal experiments have demonstrated that short-term exposure to fine particulates_,_ urban particulates, or diesel exhaust particulates, could induce inflammation and oxidative stress in peri-renal adipose tissue [24], increase cytokine expression in the kidney [25], and aggravate experimental acute renal failure [26] in rats, respectively. We speculated that there might be certain biomechanisms linking short-term PM_2.5_ exposure to the increased risk of acute kidney injury because of the marked vulnerability and high PM_2.5_ exposure of our study population, but further investigations are needed.

Numerous prior studies found that short-term elevated PM_2.5_ was associated with the risk of infections, especially acute respiratory infection [27, 28]. However, the only study that investigated the association of PM_2.5_ exposure with incident community-acquired sepsis, did not yield statistically significant results [29]. The cause for the increase in the number of sepsis cases in higher PM_2.5_ exposure groups in our study population may be iatrogenic and may be attributed to catheter-related or ventilation-related infections resulting from the more use of advanced hemodynamic monitoring and therapeutic devices due to the more significant acute severity of illnesses. Similarly, studies examining the correlation between short-term PM_2.5_ exposure and gastrointestinal hemorrhage have also failed to yield positive results [30, 31]. However, though one study reported a positive correlation between elevation in nitric oxide and an increased risk of gastrointestinal hemorrhage, the researchers admitted that distinguishing between the individual effects of nitric oxide and PM_2.5_ was challenging because they were highly correlated [30]. Hence, the causes for numerically more episodes of gastrointestinal hemorrhage in patients with higher PM_2.5_ exposure in our study may represent either an effect of nitric oxide or PM2.5. A greater number of stress ulcers related to the increased acute severity of illnesses may also be a contributing factor.

In brief, short-term PM_2.5_ exposure may increase the incidence of ANCI, a major risk factor for mortality in ICCU patients [10], and this may be mediated via some biological mechanisms in addition to its cardiovascular effect. On this basis, the complex physiological interactions between the organs or physiological systems (e.g., cardiopulmonary interaction, cardiorenal interaction, etc.) secondary to ANCI and acute cardiovascular diseases may further exacerbate the acute severity of illnesses, increase the need for advanced hemodynamic monitoring and therapeutic devices, and worsen clinical outcomes. Furthermore, the increase in emergency room visits for cardiopulmonary diseases caused by severe air pollution [32] may delay the time of first medical contact and increase the risk of mortality in patients with higher short-term PM_2.5_ exposure. All of the above may provide an explanation for the association between short-term PM_2.5_ exposure and the increased need for advanced devices and worse clinical outcomes in our study.

Our study should be interpreted in the context of the following limitations. First, PM_2.5_ exposure measurement errors were inevitable because we simply averaged monitoring results across various sites as the proxy for actual individual exposure. However, this is an inherent disadvantage of all human studies involving air pollution. Second, despite the calculation of the PM_2.5_ levels in the period of 2 days, the time of patient’s exposure to the external environment was not known. Third, potential confounders from other pollutants could not be entirely excluded because they usually correlate highly with PM_2.5_ concentration, although we found no similar association in the preliminary analysis. Forth, the interaction between ambient air pollution and weather conditions was intricate, as in previous studies, the adjustment for meteorological variables in our regression models may not exclude unmeasured cofounders. Fifth, the relatively small sample size made it impossible for us to perform further analyses to find whether some subgroup patients were more susceptible to short-term PM_2.5_ exposure. Finally, samples in this single-center study were subject to geographical restrictions, which affected their representativeness and generalization. Further high-quality multicenter time-series studies are needed to provide more evidence regarding this issue.

## Conclusion

Short-term PM_2.5_ exposure before ICCU admission was associated with an increased risk of incident major acute noncardiovascular critical illnesses and worse in-hospital outcomes in patients receiving intensive cardiac care.

## Data Availability

The datasets used and/or analyzed during the current study are available from the corresponding author on reasonable request.

## Abbreviations

GBD: The Global Burden of Disease Study
PM_2.5_: Fine particulate matter
ANCI: Acute noncardiovascular critical illness
ICCU: Intensive cardiac care unit
LOS: Length of stay
CCI: Charlson Comorbidity Index
OASIS: Oxford Acute Severity of Illness Score
DAMA: Discharge against medical advice
IQR: Interquartile range
aOR: Adjusted odds ratio
STEMI: ST-segment elevation myocardial infarction
